# Special section editorial: The frontiers of augmented and mixed reality in all levels of education

**DOI:** 10.1007/s10639-021-10746-2

**Published:** 2021-10-16

**Authors:** Ford Lumban Gaol, Ekaterina Prasolova-Førland

**Affiliations:** 1 Department of Doctor of Computer Science, BINUS Graduate Program, Binus University, Jakarta, Indonesia; 2 Department of Education and Lifelong Learning, Faculty of Social and Educational Sciences, Norwegian University of Science and Technology, Trondheim, Norway

## Introduction

As a result of the COVID-19 pandemic, the globe has seen the worst disruption of education systems in history, with schools and colleges having to shut and the number of students affected globally having increased dramatically. More than 190 nations, all continents, and over 1.6 billion learners have been impacted by the ongoing COVID-19 pandemic. More than half of the world's students are negatively affected by school closures, with 98% of lower-middle and lower-income nations bearing the brunt (United Nations, 2020).

According to UNESCO's latest statistics, as of December 1, 2020, 18.2% of total registered learners are still unable to attend school or university (UNESCO, 2020). While all of this is true, the total number of COVID-19 infections has topped 64 million instances, and over 1.4 million people have died from COVID-19 infections (3 December 2020; ECDC, 2020). When it comes to how serious a pandemic may be, and in light of the fact that many schools have decided to close for the duration of the crisis, there's a tremendous amount of pressure on education institutions, forcing them to make immediate changes to guarantee continued learning. A unique growth of e-learning took place, as remote and digital platform-based education became popular. There was a great increase in education-sector innovation because of this (Li & Lalani, 2020).

As Schleicher (2020) notes, most OECD and partner nations utilized digital distant education platforms. Such platforms contained instructional material that allowed the user to choose whether they wanted to pursue virtual meeting platforms, a variety of real-time courses in these venues, self-paced, formalized lessons, and online assistance services for parents and students. The focus of this special issue is on immersive computing technologies such as virtual and augmented reality (VR/AR) that go well beyond online learning platforms and make use of innovative new modes, that allow people to explore digital material in new ways.

A mixture of augmented reality and virtual reality has previously been utilized in a classroom setting to do research on a number of topics, including electrical and computer systems (Challenor & Ma, 2019). The learning technique based on augmented reality (AR) technology has been shown as an active learning method since they are able to quickly and efficiently convert the acquired information into long-term memory (Santos et al., 2014). Learning materials, such as video or as part of a simulation or game, are accessible for instructors to use to engage students (Wu et al., 2013). This approach may be unique in light of COVID-19's anticipated effect on a whole education system.

The goal of this special issue is to present the most recent research on VR/AR applications in educational settings, which was published within the past three years (2019–2021). All articles are divided according to time, subject, and level of education.

## A background in virtual and augmented reality

Augmented Reality (AR) systems incorporate virtual information into the user's real surroundings, creating the illusion that the information exists there (Höllerer et al., 2001). AR is the process of fusing pictures from the actual world with virtual layers of information made up of three-dimensional (3-D) models including content, photos, sounds, and videos (Vogt & Shingles, 2013). Virtual reality (VR) is generally defined as the use of a computer-generated three-dimensional world that the user can traverse and interact with, resulting in a real-time simulation of one or more of the user's five senses (Guttentag, 2010). More precisely, VR is defined by three key elements: (1) Visualization, in which the user can look around, typically via a head-mounted display; (2) Immersion, suspension of disbelief, and physical representation of objects; and (3) Interactivity, a degree of control over the experience, typically via sensors and an input device such as joysticks or keyboards). The basic difference between augmented and virtual reality is that with AR, virtual information is overlaid on a real world, while in VR, the environment is entirely simulated. Virtual Environments and Virtual Worlds are two concepts that are often used in VR research. According to Guttentag (2010), virtual reality is an experience in which the user is engaged in a virtual world. Many authors, such as (Singh & Lee, 2009) use the term "virtual environment" instead. As a non-technical word, the term "virtual environment" has a wide variety of definitions in research, from as basic as e-learning (Bray, 2002) to probably the most immersive form of VR—virtual worlds (Singh & Lee, 2009).

Virtual worlds are permanent virtual environments that are accessible 24 h a day and enable individuals represented by avatars (a three-dimensional representation of themselves) to create, play, and interact in real time (Penfold, 2009, p. 140). For example, Second Life, an internet-based virtual world where avatars interact, network, and build their own virtual places, is now one of the most active virtual world platforms (Huang et al., 2016). Second Life was founded in 2003 and now has 36 million inhabitants and over 1 million monthly active users (Linden Lab, 2013). In ten years, the virtual world economy generated USD 3.2 billion in transactions (Linden Lab, 2013).

Over the last decade, virtual and augmented reality technology has rapidly become feasible for commercial and academic applications, owing to the widespread use of head-mounted displays (HMD) and smart devices such as smartphones, tablets, and portable gaming consoles (Challenor & Ma, 2019).

AR and VR technology has numerous applications, including medicine, education, and simulated training (Yilmaz, 2016), health sciences (Moro et al., 2017), tourism (Lee et al., 2018), and navigation (Lee et al., 2018).

## The summary of the papers

This special issue provides further study on educational virtual and augmented reality (VR/AR). A large quantity of literature on immersive technology applications has been produced in different fields. The presented papers uncovered a wide range of compelling research which confirms the notion that VR/AR provides its own distinct benefits for education. The summaries of the papers are given below. However, VR/AR research for learning is in its infancy at this time. A further exploration into this area must be completed, in order to reveal the full capabilities of educational immersive technology applications.

### A paradigm shift for academia teaching in the era of virtual technology: The case study of developing an edugame in animal science

To create a virtual cattle-handling simulation (CowSim) for educational purposes, the research had two main goals: first, to record the design and construction of the CowSim, and second, to analyze preliminary survey results.

Using virtual tools like the CowSim game to improve animal science teaching is critical for the advancement of the field. So far, no other virtual cattle handling simulation game has dealt with these situations, to the best of our knowledge. To test whether students who have or do not have prior knowledge on cattle handling will have a better understanding after playing the game, we have conducted research to (1) leverage the VT (Virtual Technology) capability to develop an edutainment game on cattle stockmanship, and (2) examine if previous knowledge affects students' understanding.

Using VT, we were able to build the CowSim edugame. We found in our simulations that CowSim can't be a solo game, but things may change in the future. Our system was primarily restricted by the fact that we didn't have enough pupils, and also that the creative components we had didn't have enough capacity. CowSim's new features are part of the Capability of Creator Components (CCC), but they are complicated and were not included. This additional herd element is: a) mass addition of heifers, cows, calves, and bulls on the field at once, b) herd mentality inclusion, c) use of squeeze chutes, and finally, d) voice and body language for VR. Learning how to incorporate these changes will take time, but they will greatly improve the game's capacity to generate situations that might occur in the real world. With the benefit of 20/20 hindsight, CowSim has many drawbacks. Developing example, the development team for CowSim had very little prior expertise in game creation (UE4) and rather complex equations for animal behavioral reactions, both of which limited the game's scope. As with other enhancements, many improvements may be made to CowSim, but this needs a higher level of interaction with students and livestock experts/owners and improved software developer UE4 abilities. For a more interactive and realistic experience, several improvements are needed. Perhaps in time, the very foundations of our present academic model will be impacted by the processes of educational game production and the development of technology.

When we began the student preliminary data analysis, the results were promising, but in order to have a deeper understanding of edugames, we will have to conduct a more quantitative survey. Based on our study of the CowSim playability, the instructors of today should not be replaced by VT. This isn't all that surprising if VT gains traction. It may be that certain lecture/lab sections would cease to be useful as VT gets more popular. This is a positive development for education; although a small percentage of those who are unable to adapt will eventually wither away, the genuine instructors will flourish. The same manner our parents and grandparents were taught, we cannot teach. Every person these days has a computer in their hand, not to use it to work but to instead provide it an opportunity to reach more people and educate a larger part of the public than before.

The future seems bright. This version of CowSim does not have cumulative user tailored experiences, due to utilizing probabilistic AI. Developing edugames in the future should utilize Expert-Augmented Machine Learning (EAML), instead of parameters provided by the developer. The experience in EAML is more dynamic since it depends on how long each user plays, which affects the behavior of the animals throughout each play session. Trying out EAML in CowSim would offer a better experience customized via trial and error for learning how to respond to the actual world rather than learning action and response parameters that are already set in stone. More lifelike experiences may possibly be provided by the capacity of the AI to learn and adapt.

This assessment would focus on how many animals fall during the interaction, how many cattle vocalize, how many animals are moved with additional equipment (such as a cattle prod), how many animals refuse to move forward, how many animals turn their backs while being handled, and how many animals back up in a single file. The CowSim model was created as an evaluation tool for a professor to gauge the user experience for his lectures. Student help will no longer be required in the future versions of CowSim that make use of EAML. It is best if the experience itself is measured via machine learning performance evaluation to quantify performance and handling effectiveness.

### Developing augmented reality-based learning media and users’ intention to use it for teaching accounting ethics

Fraud is growing in corporate operations, and thus ethics education is more important in business schools. It's also difficult to attract and hold the attention of millennials and Zers, who are often digital-savvy, while teaching concepts of accounting ethics. For millennial and Z generation students, one of the appropriate learning medium is augmented reality technology, which incorporates both the actual world and the virtual world to display a replica of the real environment.

The project's primary goal is to design Augmented Reality-based instructional material to help teach young adults and the Z generation how to be more ethical with the use of accounting. In addition, this research seeks to answer two related questions: first, does faculty interest in using the application rise, and, second, if interest rises, are participants' intentions influenced by perceptions of ease of use and utility? Using augmented reality apps would boost the learning process as it allows them to serve as interactive case learning and prevents users from becoming bored. This project utilizes the SCRUM approach for the creation of the media. SCRUM divides tasks into four stages: The Product Backlog, Sprint planning, the Sprint Backlog, and the Sprint Review. The System Usability Scale (SUS) is used to assess how much the user thinks the system is usable. The ultimate SUS score results in a 90.27, which indicates that the application is very suitable for usage. The results show that the app users intend to use the application, which is linked to their perception of the program's ease of use and their perception of the application's usefulness. The quality of the AR application created in this research and/or the development of additional AR-based apps utilizing different instances is likely to be further improved in the future. Additional studies may potentially result in more interactive learning media, such as the one used in this research, as well as others. New study may now examine if AR-based ethical learning material include interactive elements, and if so, how it influences student cognitive style and attitudes.

### Evaluating the attitude towards the intention to use ARITE system for improving laboratory skills by engineering educators

The main purpose of laboratory skills in engineering education is to foster graduate characteristics in students. These characteristics, including as problem solving, design, teamwork, and engineering tools, may be obtained via a hands-on approach that focuses on experience. Thus, in order to enable engineering graduates to become effective teaching and learning laboratory technicians, there are substantial barriers that must be overcome. Though the adoption of new teaching–learning pedagogical technologies over the past three decades has led to new tech-enabled teaching–learning applications that may be used in and outside of the classroom, it has been noted that augmented reality (AR) has emerged as a new technology in the education industry, which may re-shape the educational system. The design and development of an AR-based learning system to make learning effective and efficient is still ongoing. Many AR-based learning systems have been created in many fields including architecture, mechanics, electrical machinery, electronics engineering, physics, chemistry, design, and mathematics. In this research, the goal is to create an AR-based learning system, namely an ARITE system, to help first-year engineering students to understand embedded systems. Developed AR application EmbedAR provides a seamless transition from the hardware abstraction level to the application development level, which offers a smoother transition from the hardware abstraction level to the application development level. Augmented reality is a good fit because it offers the imagery and a feeling of connection with the information, which users will learn via interactive learning and activities, and guarantees successful and productive learning. The behavioral intention of teachers to utilize the ARITE system during their courses must be assessed after designing and implementing the system. The ARITE system includes four dimensions: perceived utility, behavioral intention, convenience of use, and attitude towards usage.

This research statement is the foundational statement from which research hypotheses are developed in the introduction and outcome sections of the article. Thirty-four electrical and communication engineering faculty members with an average of 10 years of teaching experience participated in this research. Following a demo session, the faculty members had a hands-on session where they learned about the ARITE system. In order to collect detailed information, a questionnaire was administered including 12 questions distributed on a seven-point Likert scale, with seven being the midpoint of each scale point. The data obtained was analyzed using IBM SPSS statistical program. Based on the findings, it is reasonable to conclude that and no positive relationship could be discovered between ease of use and usefulness (PEU—PU) and attitude and behavioral intention of using the ARITE system (AT—BI). However, following thorough examination, some acceptable theories had a notable beneficial effect on results. Usability has a positive influence on both attitude and behavioral intention (PU—AT). In turn, user-friendliness also has a positive influence on attitude and behavioral intention to use the ARITE system (PEU—BI). This overview summarizes the findings, showing that using the ARITE system in normal classroom settings offers a beneficial contribution.

In the current system, three learning activities were built for, which may be improved or expanded. DC motor control, actuator control, sensor interface, and actuator control may also be included in future designs. Using the same method on students, the next stage would be to determine how much pupils have learned, what motivates them, how confident they feel, and how intelligent they are using ARITE. EmbedAR may be modified into a web-based AR application that provides broad learning coverage and enhanced student assessment and feedback capabilities.

### Generative learning strategies do not diminish primary students’ attitudes towards augmented reality

This paper looks at a timely new issue in the world of educational technology research and evaluates the value-added effects of two mobile AR learning environments.

As part of an AR (augmented reality) experiment, a total of 56 primary school children participated, either in an experimental group or a control group. An experimental group using augmented reality (AR) together with other methods based on generative learning theory, namely self-explanation and self-testing, learnt the experimental concepts. In the control group, just AR was used. Positive views regarding AR as a learning tool were examined whether or not adding learning methods had an effect on this. Compared to the control group, the experimental group of students had similar favorable views regarding AR as a learning tool. On the other hand, the results revealed substantial variations in the level of skepticism shown by students who learned using both AR and learning methods. It was discovered that men rated AR technology as being simpler to acquire outside of the classroom as well. The findings presented have implications for theory and practice as well as future study.

This research's most notable contribution is that views about using AR for learning in elementary school are highly favorable, and the introduction of learning methods has no impact on that. No other gender differences were discovered, just those associated with accessibility. This is because boys have more confidence in their computer abilities, which in turn makes them more likely to explore other avenues of information. When it comes to studying using AR, females often found it more difficult. This certainly has to be studied further and incorporated into real-world applications. It is advised to become familiar with the application ahead of time by doing some preparatory training.

Although this work contributes significantly to a newly relevant research field, the findings given here should be seen as a foundation. Additional factors, content, and AR kinds are recommended for further study. In reality, this study shows how elementary school children see AR as a medium that is both fun and educational.

### Learning basic concept of computer programming with path-finding task in AR and its properties

This article creates and installs a teaching software for absolute novices as well as those who are completely new to programming. Due to prior knowledge about variables, their types, operators for arithmetic calculations, and relational calculations, traditional computer programming learning systems require students to already know about variables, their types, and operators for addition and multiplication, thus introducing a great deal of extra burden to the learners. A focus on sequential, conditional, and iterative controls allows for not requiring prior knowledge and involves a path-finding task that confines the representation of program codes to a specific task that is a navigation task on a Manhattan grid map.

A system architecture was described and an experiment was performed to test the hypothesis about education feasibility, with three possible outcomes. Our findings indicate that our approach has the potential to help novices learn how to create code for longer programs, since the ratio of longer programs to shorter programs fell from 2.186 to 1.267 independent of AR competence. Results show that our system leveraged the motivation of learning by raising beginners' score from 5.00 without AR capability to 6.67 with it for interest, and from 5.67 to 7.00 for amusement.

To date, this article has had two significant contributions to computer programming education. In the first instance, the introduction of a new learning strategy for understanding the three control flows is established via the use of a path-finding assignment. Combination of such elements and their potential effect on comprehension of program codes has been addressed in this article from a number of distinct perspectives. The second factor is how augmented reality (AR) impacts motivation for learning. Credible sources assert that tangible user interfaces, which particularly catch the interest of youngsters, act as effective learning tools. This article incorporates an exploration of the connection between augmented reality and learning motivation, and also showcases the results of this work.

### Problem-based gaming via an augmented reality mobile game and a printed game in foreign language education

In this study, the researchers aimed to uncover if problem-based gaming, which is currently carried out on paper or with immersive technologies, is conducive to comparable levels of student involvement and positivity. According to the findings, regardless of whether the education took place in an English medium, Korean EFL (English as a foreign language) learners demonstrated strong levels of behavioral, cognitive, and emotional engagement, as well as positive attitudes, so long as it was done according to the principles outlined in GBL and PBL. Students perceived EFL instructional methods to be less effective when compared to the benefits that AR platforms offered, especially when it came to immersive and genuine language learning.

This research does have certain shortcomings. Despite this research's likely being one of the first to compare an AR mobile game with a print-mode game in L2 education, this study can't prove the two distinct approaches to game use have differential impacts on language abilities. To further understand the impacts of mobile games on particular foreign language abilities, future research should create a series of AR games, apply them in long-term teaching, and observe their effects on various school levels. A location-based AR mobile game may be affected by the social environment or culture in which the game is situated. However, in the present research, location-based AR mobile games were not examined from a social and ecological perspective. Future studies anchored in sociocultural frameworks may provide fresh views and concepts on AR mobile games focused on locations.

Despite the limited capabilities, the current investigation has significant consequences for technology-based second language (L2) teaching. The following game design and development methods provided here may be very valuable to L2 instructors and researchers as they apply for grant money to build a narrative-driven, location-based mobile game. This case study shows that AR location-based mobile games may be used to foreign language settings, providing students with chances to communicate in natural writing via case reports. AR mobile games provide many situations or contexts for real-world communication with a strong sense of immersion and authenticity, which helps their use in EFL settings.

This research therefore provides as an example for curriculum architects and instructional designers, who may include augmented reality mobile games into foreign language learning programs or modules. One of the newly emerging findings regarding the efficacy of emerging technology in language acquisition is that the main factor is not the use of new technology but rather creative teaching practices in second language education. The study found that utilizing cutting-edge technology may not always lead to greater student involvement or L2 learning attitudes. This study offers a platform for future investigation as well as providing a unique viewpoint on technology-assisted teaching already in place.

### Running an XR lab in the context of COVID-19 pandemic: Lessons learned from a Norwegian university

Universities and businesses were unprepared for the adjustments necessary to contain COVID-19's expansion in Norway and worldwide. Universities were forced to make an abrupt transition to online education. There is considerable ambiguity about how pandemic-control efforts will impact universities in the near and medium term. Such measures will have an effect on how research is conducted, which is often based on students, researchers, and volunteers using equipment and apps in XR labs.

Given the present state of affairs, it is probable that XR researchers will be unable to run their labs in the manner they did prior to the pandemic, even if limitations are eased. As a result, the authors of this paper created a novel approach for operating an XR lab for research, teaching, and dissemination purposes, complete with stringent hygiene measures to ensure the lab's users' trust and safety. These rules may also be applicable to other university-based XR laboratories. While the epidemic wreaked havoc, it also sparked ingenuity. Immersive technologies have the potential to provide rich distant communication and collaboration, as well as education and research. Numerous courses, seminars, and conferences have been relocated to online virtual reality platforms such as Virbela and AltspaceVR in recent months.

These features could be investigated further in the future, for example, to assist research activities when researchers need to take the equipment home, by providing remote help to peers. The authors are now doing research on how to use XR to enable rich and interactive university education in pandemic and post-pandemic settings, particularly in practical areas where online lectures are insufficient. Additionally, the authors have developed a new course on XR for remote collaboration to assist recently jobless individuals in adjusting to their new normal and working realities.

### User acceptance of augmented reality welding simulator in engineering training

The use of augmented reality (AR) into welding training is expected to improve operational efficiency, security, and productivity while lowering consumable and infrastructure costs. Prior research has explored the integration of augmented reality simulation into applications such as medical procedures or aviation, highlighting the need for these systems to be more usable. However, research on integrating augmented reality into welding instruction is still restricted. It may assist novice welders in learning efficiently and preparing them for employment in the industry. The research was motivated by the continuing usage of augmented reality in manufacturing training, and its uniqueness stems from the examination of the most important variables influencing actual augmented reality system use. The primary aim of this study is to assess the application of augmented reality technology in welding instruction by extending an innovative model to account for pedagogy and technology. The purpose of this article is to investigate and comprehend the variables connected with AR welding training that may result in target achievement and, therefore, influence users' decision.

This research used a modified Technology Acceptance Model, which incorporates two additional variables (reported pleasure and system quality), and a sample size of 200 trainees. External factors are shown to be significant determinants of perceived usefulness and convenience of usage. The desire to utilize an augmented reality simulator is closely related to the system's quality and perceived ease of usage. The results assist augmented reality developers in improving the quality of augmented reality simulation training systems in order to improve users' experience and behavioral intention to utilize them.

Traditional welding training is expensive in terms of consumables and equipment, has physical hazards, and emits hazardous gases. On the other hand, AR welding simulators pose no physical hazards or emit harmful gases and result in substantial cost and workshop time savings. This is the first comprehensive experimental study to evaluate the acceptability of an AR simulator in a real-world engineering welding teaching setting.

The present study produced some intriguing research findings; nevertheless, it has significant drawbacks. To begin, the population comprised practitioners with the lowest welding level who participated in the training utilizing the AR welding simulator. A future study may include experts at the highest levels, such as engineers and technicians, to replicate the research method and validate the findings.

### Utilizing social virtual reality robot (V2R) for music education to children with high-functioning autism

Virtual Reality (VR) technology is a rapidly expanding area of study that has been used to a variety of disciplines including psychology, education, and therapy. The purpose of this research was to assess the feasibility of incorporating virtual robots into active music instruction sessions and to determine the efficacy of utilizing virtual reality robots in intervention sessions. The encouraging findings from this preliminary research suggest that virtual reality technology may be beneficial in the rehabilitation of children with autism spectrum disorders.

The findings of this research indicated that children's capacity to perform musical phrases was substantially different throughout the second half of the sessions than during the first half. Additionally, by categorizing the activities as tough or easy, we discovered a substantial difference in the subject's performance on difficult tasks between the first and second halves of the sessions. However, because to the ceiling effect, there was no significant change in basic tasks. This significant difference, combined with the evaluation of the children's performance during the sessions via video coding and an automatic assessment method, demonstrates the effectiveness of virtual reality technology in improving the ability of children with autism spectrum disorder to play musical phrases.

Nonetheless, we observed no substantial increase in the cognitive abilities of the children included in this study after the occurrence of a ceiling effect throughout the educational program. Additionally, we found that each child's performance varied considerably. Our research implies that we were able to distinguish between individuals with varying degrees of autism, which is another significant accomplishment of this research.

### Writing an expository text using augmented reality: Students’ performance and perceptions

Writing is a difficult job for students studying English as a Foreign Language (EFL). Students are not provided with adequate chances for practice in the classroom. As a result, they are unmotivated to write or like writing in English, and they struggle with planning, organizing thoughts, expressing a clear goal, and selecting suitable words to convey their ideas while producing English writings. By incorporating modern technology into teaching, such issues may be mitigated. The primary objective of this research is to determine if the usage of augmented reality materials can aid high school students in the process of creating English texts.

Additionally, the research aims to elicit students' perspectives on the usage of augmented reality. The research is designed in a quasi-experimental fashion. Two classes of high school students with a B1 level of English proficiency participated in the experiment. The study enrolled a total of 48 students. The courses were divided into experimental (*N* = 24) and control (*N* = 24) groups in accordance with the design of the AR-based materials. During the same time period, both groups worked on the identical subject. After students completed their compositions, their views of the usage of augmented reality-based elements were elicited through a questionnaire. The statistical analyses revealed substantial disparities in the groups' writing scores.

The results showed that the usage of augmented reality-based materials had a moderate impact on the students' chosen writing abilities. Additionally, the findings indicated that students had a favorable opinion of the AR-based writing experience. The results indicate that augmented reality-based writing exercises enhance students' motivation for writing, which may result in improved performance.

Two major conclusions may be made from our results and conversations. To begin, students in EFL settings feel demotivated when assigned writing assignments, partly owing to inadequate language resources and partly due to learning situations that offer insufficient assistance. We predicted that by incorporating modern technology into instructional methods, these issues might be minimized. As predicted, using AR-based writing experiences with B1 proficiency level high school students not only cultivated good attitudes, but also improved their writing abilities. AR-experiences, such as the one developed for this research, enabled students to learn new material in a fun and engaging manner.

## Conclusion

As we can see from the presented papers, VR and AR technologies in most cases provide an engaging and motivating educational experience, proving to be a valuable supplement for traditional teaching methods and tools, especially in a post-pandemic context. The efficacy of immersive technologies in education and the corresponding learning outcomes compared to other mediums and tools (such as textbooks and 2D online platforms) depend on several factors and require further investigation. These factors include an appropriate choice of educational content, optimization of the content for the chosen medium, availability of suitable XR applications, pedagogical methods used, existing technological infrastructure and support provided at educational institutions, technology acceptance and integration of immersive technologies into the existing educational practices, just to mention a few. The COVID-19 pandemic has highlighted the need for innovative approaches supporting hybrid teaching and learning. Immersive technologies will play an important role in the educational landscape of the future by bridging and connecting physical and virtual learning environments.
